# Diseases of Children

**Published:** 1913-09

**Authors:** 


**Diseases of Children.** By Various Authors. Edited by A. E. Garrod, D.M., M.A., F.R.C.P., F.R.S., F. E. Batten, M.D., M.A., F.R.C.P., and Hugh Thursfield, D.M., M.A., F.R.C.P. Pp. xv, 1184. London: Edward Arnold. 1913. 30s. net.

We live in an age of encyclopædias, and medical literature has fallen in with the prevailing fashion. It would almost seem as if knowledge were becoming so extensive that no one could pretend to more than a general acquaintance with any subject, and that compressed works of reference, wherein special points may readily be looked up, and then perhaps forgotten, were the only means by which ordinary persons could keep abreast of the times.

The present volume on *Diseases of Children* is certainly a marvel of compression, without any undue sacrifice of lucidity. Pediatrics, as transatlantic authors love to call it, is a specialism of a peculiar character, in that its range is almost co-extensive with that of general medicine and surgery, and it is somewhat significant that nearly all the writers in this volume, while possessing special knowledge and experience in children's diseases, are also on the staff of general or special hospitals where adults form the bulk of the patients, and are known in general medicine and surgery as men of light and leading.

The names of the editors are, perhaps, a sufficient guarantee of the excellence of the contents, and their own contributions form a series of monographs of sufficient value to justify the book were there nothing else in it to commend. Dr. Batten's account of organic nervous diseases is particularly excellent, and gives a clear and well-arranged account of a complex subject in a most succinct manner. The diseases of the alimentary system, by Dr. Hutchison and Dr. Waugh, and the feeding of infants and children, by Dr. Cautley, are also very valuable chapters.

Dr. A. E. Garrod contributes a short and very philosophical chapter on the general subject of disease in children, and also writes on the diseases of the ductless glands and disorders of metabolism; the latter chapter is specially interesting as containing a *résumé* of much of the author's original investigations. The chapter on immunity, serum therapy and vaccines, though in itself a useful exposition of the subjects, does not appear to have any special bearing on disease as it is seen in children, but in other respects the book keeps well within the range indicated by its title. The apportionment of space to the various subjects has been well thought out, and while rare conditions and obscure points are duly included, the practitioner will find that more ordinary matters are allowed sufficient space for full discussion, a fact which will render the book of considerable practical value to the average man.

It is impossible in reviewing a work of this sort to criticise special points, but it may be said generally that while the views set forth by the various contributors are sufficiently individual, they represent the highest standard of medical thought in this country, and may be accepted as authoritative, though in details, perhaps, there may be differences of opinion.

We can confidently recommend this book as a very valuable work of reference, which should prove most useful to all practitioners who have to treat disease in children. It is up to date, practical and clearly written, and considering the size of the volume and the large field covered by its subject-matter, remarkably cheap.

